# From dreamers to doers: Navigating the doctoral journey in family medicine and primary care

**DOI:** 10.4102/phcfm.v17i2.5192

**Published:** 2025-11-09

**Authors:** Klaus B. von Pressentin, Mpundu Makasa, Akim T. Lukwa, Innocent K. Besigye

**Affiliations:** 1 Division of Family Medicine, Department of Family, Community and Emergency Care, Faculty of Health Sciences, University of Cape Town, Cape Town, South Africa; 2 Department of Community and Family Medicine, School of Public Health, University of Zambia, Lusaka, Zambia; 3 Department of Family Medicine, School of Medicine, Makerere University College of Health Sciences, Kampala, Uganda

**Keywords:** PhD journey, primary care research, doctoral experiences, education, graduate, primary health care, Hero’s Journey, African PhD graduates

## Abstract

This article examines the transformative journey of pursuing a Doctor of Philosophy (PhD) in family medicine and primary care through the lived experiences of four African scholar-practitioners. Using the Hero’s Journey framework, the authors reflect on the emotional, intellectual and structural aspects of doctoral education, highlighting the unique challenges faced by clinician-researchers in resource-limited settings. Each vignette illustrates the transition from dreaming to doing by navigating identity shifts, funding obstacles, methodological complexities, as well as the need to balance clinical service with academic development. The article offers practical insights for prospective doctoral degree candidates, including the importance of defining one’s purpose, building supportive networks, and adopting adaptable strategies. It also calls for institutional reforms to enhance supervisory capacity and funding mechanisms. By merging personal narratives with reflective analysis, the authors aim to inspire and equip future doctoral candidates in family medicine and primary care, encouraging them to view their journey not just as an academic endeavour but as a pathway to leadership, thereby strengthening the discipline’s knowledge foundation and enhancing primary care. This contribution serves as a guide for moving from aspiration to action, offering practical wisdom for navigating the complexities of doctoral education in African primary care contexts.

## Introduction

Despite the growing need for established researchers, especially in sub-Saharan Africa, which contributes only 1% of global research output, the doctoral journey remains daunting for many.^1^ This doctoral degree is viewed as a significant investment and its educational purpose differs from a Master’s degree (which can be one of three types, namely taught, research or professional Master’s). In family medicine, a professional Master’s degree is designed to educate and train graduates for advanced professional employment as specialist family physicians. A professional Master’s degree typically includes a mini-dissertation describing a single research or technical project. In contrast, a doctoral degree or a Doctor of Philosophy (PhD) requires a candidate to undertake research at the most advanced academic levels, culminating in the submission, assessment and acceptance of a thesis. Higher education frameworks have defined the doctoral degree as requiring a demonstration of high-level research capability and a significant and original academic contribution at the frontiers of a discipline or field.^2,3^ The doctoral work can be discipline-based, multidisciplinary or applied research, requiring at least 2 years of full-time study, typically following completion of a Master’s degree. Doctoral degree graduates are expected to possess the capability to supervise and critically evaluate the research of others within their fields of specialisation.

High attrition rates and emotional strain discourage potential candidates, even as institutions hope that students will find the process fulfilling. A significant number of doctoral students, in some cases as high as 70%, never complete their degrees.^4^ Studies have also shown that one in every three students contemplates dropping out at some point.^5^ This occurs despite substantial investment of time, money and energy by the doctoral students and their supervisors. Several factors can significantly impact the journey towards completing and attaining a doctoral degree. Reasons or factors may lie within and beyond the student’s control, including sociodemographic characteristics of PhD candidates, financial hardships, limited resources and training, the quality of supervision and the coping strategies employed by those undertaking a PhD programme.^6^ The student must learn adaptive mechanisms and techniques to address these obstacles. Van Rooij et al.’s study on doctoral success provides valuable guidance, which emphasises the importance of supervision quality, psychosocial factors, workload, autonomy, a sense of belonging and alignment with supervisors’ research.^7^

The evolving role of family medicine and primary care in healthcare systems underscores the growing importance of developing researchers and academic leaders in the discipline. Family medicine PhD students often occupy unique positions as clinicians, educators and researchers. Despite their increasing presence, there is a limited body of literature capturing the lived experiences of family medicine PhD students. Generic guides, such as Brennan’s ‘*100 Rules of PhD*’, offer useful advice but often lack the lived experience and contextual nuance needed to support students.^8^ Resources that integrate the emotional, intellectual and practical aspects of the doctoral journey are scarce, and many students face the dual-identity challenge of balancing their roles as clinicians and researchers.

## Why this guide matters

This article aims to provide a reflective, supportive and practical guide for navigating the PhD journey, drawing on the authors’ lived experiences. It seeks to normalise the highs and lows of the PhD experience while inspiring and equipping future candidates and guiding supervisors. We would like to remind readers that embracing the complexity of family medicine doctoral journeys differs from those in other disciplines and even within the same discipline (e.g. dual clinical–academic roles, decentralised and rural settings and a low supply of experienced doctoral-level supervisors).^9^ We have written this contribution for the *African Journal of Primary Health Care & Family Medicine* (PHCFM) special collection with a specific audience in mind: those colleagues who are dreaming of pursuing a PhD and would now like to find out how best to transition from dreaming to doing.

## Introducing the approach to unpacking our stories

To support prospective African PhD candidates in family medicine and primary care, we adopted a reflective narrative approach based on the Hero’s Journey Framework, as conceptualised by Joseph Campbell and applied to doctoral education by Salter.^10,11^ Salter aligns the three main acts – departure and call to adventure, initiation along the road of trials and the return – with key doctoral phases: admission into doctoral education, life as a doctoral student and dissertation completion. Drawing on Salter’s adaptation of Campbell’s Hero’s Journey, we use six archetypes – Innocent, Orphan, Wanderer, Warrior, Altruist and Magician – to frame the evolving psychological challenges of doctoral education. These archetypes reflect different stages of transformation: the Innocent begins with naive optimism and trust in the system; the Orphan enters the journey out of necessity, carrying emotional burdens; the Wanderer seeks independence and meaning amid uncertainty; the Warrior confronts obstacles with courage and determination; the Altruist is driven by a desire to make a meaningful impact while balancing passion and objectivity; and the Magician, embodying the culmination of the journey, integrates the strengths of all previous archetypes to become a wise, independent scholar capable of mentoring others. Together, these archetypes offer a reflective framework for understanding the identity formation and transformative nature of doctoral education.

To bring these archetypes to life, we share four personal vignettes (Box 1 – Box 4), each structured around the early stages of the Hero’s Journey: the Call to Adventure (beginning the PhD); Crossing the Threshold (major challenge); and Trials and Allies (supervisors, peers and tools). These stories reveal how identity, purpose and resilience evolve through the doctoral process.

BOX 1 Vignette 1: Navigating a taught PhD abroad.

*My call to adventure*

My professional journey started with an internship and a senior house officer position at a tertiary hospital, followed by a mandatory rural placement in a primary care setting. After completing my Master’s in Public Health, I worked both as a clinician and a public health practitioner, eventually becoming a district manager responsible for programme planning. Despite nearly 8 years of experience, I found it challenging to identify a clinical speciality that matched my interests, especially in family medicine, which was not available in my area. To enhance my education, I pursued a PhD in Epidemiology at the University of Bergen in Norway.

*Crossing the threshold*

I pursued a taught PhD that required coursework. After arriving in Bergen, I shifted from a management role in planning and development to being a student. Alongside coursework, I developed my PhD proposal, balancing work and study. I managed my time to handle courses, reading, research and writing. Regular meetings with supervisors and weekly research seminars helped track progress. Listening to peers motivated me and kept me on pace.

*Trials and allies*

Embarking on the PhD journey soon made me realise that I was a ‘senior student’, responsible for demonstrating my ability to work independently and manage my own progress. I was expected to organise meetings with my supervisors and share my work in advance. These meetings focused on specific sections of my research, allowing for discussions that clarified methodology and project details. After completing our coursework in the first year, I experienced feelings of loneliness as we transitioned to working individually in our offices. Each of our research plans varied significantly based on personal interests and locations, leading to different timelines for fieldwork and graduation. Our close-knit group began to feel disintegrated because of the unique demands of our projects.

Key factors in my success included a fully funded scholarship that covered living expenses and research costs, reducing financial stress. However, I needed extra funding for laboratory work, such as Polymerase Chain Reaction testing. Having dedicated supervisors who provided prompt feedback helped me stay on schedule and graduate on time. The programme also allocated funds for academic materials, giving access to essential resources. In addition, milestones such as conference attendance and presentations were supported by these academic funds. Training in a well-structured PhD programme was invaluable, especially because my home country lacked a School of Public Health at the time, making such studies impossible locally. I appreciated that the programme was flexible and allowed me to travel home as often as reasonably possible. Despite the demands of school, this helped me maintain a healthy balance between my school and personal life.

Writing can be daunting, especially for beginners who struggle to conceptualise topics. My focus shifted considerably after discussions with my supervisors, which, although sometimes frustrating, were crucial for pinpointing the right area, formulating a relevant question and closing knowledge gaps. Moving in a new direction required me to familiarise myself with general medicine, but I managed to do this quickly. Along the way, I gained skills in literature searches, writing and responding to feedback from ethics committees and journal reviewers. Using different study designs and data collection tools helped me learn various methods, while managing fieldwork taught me the importance of patience and teamwork. Publishing my first academic article was particularly challenging, but it marked a significant milestone in my PhD journey. Having a supportive team of supervisors, including a senior professor and a recent PhD graduate, was invaluable. Their dedication and commitment greatly supported my academic, social and mental well-being.

PhD, Doctor of Philosophy.

BOX 2 Vignette 2: Balancing clinical roots and academic purpose.

*Call to adventure*

At the beginning of my PhD journey, I was a newly qualified family physician working at a rural district hospital, where I balanced clinical duties with student teaching. The healthcare system was still unsure about the role of family physicians, and I was trying to establish my place. When the opportunity to join a national research project and pursue a full-time PhD to explore how family physicians can strengthen district health services arose, I felt a strong pull – but I couldn’t quite put my finger on why.

During my interview for the PhD position linked to the funded project, for which my supervisor was the principal investigator, I was asked about my ‘why’. I remember honestly replying: ‘I just know I need this opportunity’. It wasn’t a polished answer, but it was true. The sense of purpose became clearer later. Arthur Brooks describes this well: ‘A rhumb line (used by sailors to simplify charting and navigation by providing a fixed course) is a path of constant bearing – it doesn’t always get you there fastest, but it keeps you true’.^12^ The idea of remaining faithful to a deeper goal, even when the destination is unclear, resonated with me.

*Crossing the threshold*

The first challenge was negotiating a hybrid model: the project role funded my time, but I wanted to stay clinically active (1–2 days/week) and remain within my rural community. This involved developing an agreement with my supervisor to allow me to spend 3–4 days per week in Cape Town at the university and the remaining days at home supporting my rural district hospital as a clinical family physician. A senior colleague advised: ‘Stay clinically connected – it will keep your research grounded’. That became a guiding principle. Another colleague expressed surprise at my decision to step away from the expected path towards a family physician consultant role, saying, ‘You’re moving away from what you were trained for’. But I saw it differently: I was investing in a broader contribution to the discipline and suspected that my path would lead towards academia.

*Trials and allies*

Early hurdles included navigating methodological considerations (which study designs will be ideal to capture the early impact of our discipline in strengthening the South African district health system), ethics processes (obtaining ethical and gatekeeper permission for fieldwork across seven South African provinces) and imposter syndrome (‘Am I the right person to take on this vital role to study the early impact of my discipline?’ – pivoting between the emic and epic, or insider and outsider perspectives). My supervisor was a reliable guide in helping me overcome these challenges. I also appreciated the support of close colleagues, including the fellow project colleague who mentored me during this early phase of my PhD as a near peer while completing her own PhD. Despite having no formal coursework requirements, I was able to participate in opportunities such as the African Doctoral Academy, which helped me enhance my skills in using software for quantitative and qualitative analysis methods. Rural life itself provided space for reflection. The balance between clinical work and research helped me stay aligned with my purpose.

PhD, Doctor of Philosophy.

BOX 3 Vignette 3: Persevering through scarcity and self-doubt.

*Call to adventure*

My professional career as a doctor has mainly been in academic medicine. Therefore, doing a PhD was not an exception but a necessary part of my career journey. However, there was no opportunity to undertake doctoral studies without switching my speciality from family medicine and primary care, as the required mentorship and supervision were not available in that field. The available opportunities were in other specialities. I was not prepared to join new networks and disciplines. Another challenge was the lack of funding.

As a result of my strong desire to pursue a PhD, I continued to seek support and funding. I regularly shared my aspirations with many colleagues, including juniors, peers and seniors within the family medicine community. This curiosity was noticed by colleagues who could mentor or supervise a PhD and those with access to funding. This recognition marked the beginning of my journey.

*Crossing the threshold*

Once supervision and funding were secured, the preparation began. Several challenges arose during this phase. The major hurdle was the scarcity of faculty staff in my department who, because of heavy workloads, found it difficult to grant study leave for dedicated doctoral research time. Family issues also arose regarding how to continue meeting my obligations to those under my care. Furthermore, I experienced anxiety from the uncertainty about what the journey would entail and how it would unfold. Finding a research topic proved to be difficult, although reviewing prior research studies was helpful. Inadequate funding was another obstacle; the only support available covered tuition fees, not research expenses.

*Trials and allies*

Once the journey began, new challenges emerged, including balancing work and study simultaneously. Strategising my time between work and research helped a great deal. Some days and times were dedicated to work while others were reserved for research. The colleagues within my department understood the precarious circumstances I faced and reduced my teaching load. For example, I was less involved in undergraduate tutorials.

Difficulties with obtaining research funds also arose. With my supervisor’s help, I continued to apply for research funding but remained unsuccessful. Then, an opportunity arose to apply for research funding from the Ugandan government through my university. I applied and succeeded. Problem was solved. However, it was only for 1 year, with a 1-year no-cost extension. Having an already approved proposal was helpful, as I was ready to begin the research work.

Technology proved to be a great ally. I was in my home country, but had regular online Zoom meetings with my supervisor, even before Zoom became commonly used during the coronavirus disease 2019 (COVID-19) pandemic. I also participated in some workshops offered by the university’s postgraduate office via Microsoft Teams.

Procrastination and writer’s block were regular challenges. I had to find ways to deal with these two obstacles. A PhD involves reading and writing – both tasks that can be somewhat uncomfortable. Using online resources and coaching, such as Daphne Gray-Grant’s writing courses, was very helpful.^13^ Online courses, such as the Massive Open Online Course (MOOC) by Barbara Oakley on learning how to learn, have also proven beneficial.^14^ A PhD can be a lonely journey. Having a study companion is extremely helpful. I was fortunate to have a colleague in the department who was also on the same journey. We shared our challenges almost every day. Sleep is a vital ally for a PhD student. Adequate sleep helps maintain a fresh mind, enabling one to think creatively and critically. Adjusting my work schedule to ensure enough sleep was important. Consistency is also a key ally. Despite all the trials encountered, continuously thinking and working on PhD tasks is crucial.

Taking a break from the PhD for more than a week can lead to undesirable effects, making it difficult to get back on track and costing valuable time. Developing an open mind is essential if you do not yet possess one. The PhD has many stakeholders, including your supervisors, journal editors and reviewers, examiners and others. The likelihood of encountering divergent views from any of them is high. Be receptive to change, criticism and differing opinions. Personal patience and resilience are vital allies. Many aspects of the PhD journey – such as the time taken for Institutional Review Board approval or the turnaround time for manuscript submissions – may be beyond your control. These delays can cause significant anxiety, especially given the tight timelines set by universities and institutions.

PhD, Doctor of Philosophy.

BOX 4 Vignette 4: Transforming purpose into scholarly impact.

*Call to adventure*

When I began my PhD, I quickly realised that knowing my ‘why’ wasn’t just helpful; it was essential. A PhD is not simply an academic exercise; it’s a marathon of critical thinking, problem-solving and resilience. There are moments when the excitement of a new idea gives way to the grind of data collection, the frustration of unexpected results, or the sting of critical feedback. In those moments, my ‘why’ became my compass. It reminded me that I wasn’t just chasing a degree; I was working towards knowledge that could influence policy, improve lives and challenge the way we think about health and inequality. That purpose gave me perspective, turning obstacles into stepping stones rather than roadblocks.

*Crossing the threshold*

I have met people who began their PhD because it seemed like the logical next step, but without a strong ‘why’, it’s easy to feel lost or disconnected from the work. For me, my ‘why’ shaped the research questions I asked, the collaborations I pursued and even the sacrifices I was willing to make. It kept me grounded in the bigger picture, ensuring that every late night in front of Stata, every draft revised, and every conference presentation was a contribution to something that mattered deeply to me.

*Trials and allies*

One of the key lessons from my PhD journey so far has been the importance of managing expectations of both myself and the process. Initially, I started with great energy and enthusiasm, but I quickly realised that timelines in academia are often flexible. Supervisors, while supportive, often juggle multiple commitments, which can result in longer turnaround times for feedback. This realisation was initially disheartening, but it taught me to proactively plan around these delays (e.g. setting earlier internal deadlines, scheduling follow-ups or using waiting periods for tasks like literature reviews).

Another unexpected challenge was the administrative burden of the PhD, especially when coordinating data collection. Ensuring timely payments for research assistants, troubleshooting fieldwork logistics and maintaining team morale required meticulous organisation. For those pursuing a thesis by publication, the current journal turnaround times add another layer of complexity. Some institutions require all articles to be accepted before submission, so building buffer time into the timeline (and considering preprint options or parallel submissions where permitted) is crucial.

Beyond logistics, the PhD can be a profoundly isolating experience. Unlike structured coursework programmes, there’s no built-in cohort, and few people truly understand the nuances of your research, which means well-intentioned advice sometimes misses the mark. This isolation is especially acute for those joining a new institution without existing connections. To counter this, I’ve learned the importance of seeking out mentors (both within and outside my field) and nurturing peer networks, even informally. Discipline and time management are essential, but so is self-care. It’s easy to become absorbed by the PhD, but the degree is temporary; relationships, however, are not. I’ve seen colleagues lose touch with family and friends, only to regret it later. Setting boundaries (e.g. dedicated ‘off’ hours, regular social plans) and remembering that the PhD is just one part of life have been vital for my well-being.

PhD, Doctor of Philosophy.

## The stages of the PhD journey

The narrative arc outlined in our vignettes emphasises the PhD as a transformative journey rather than a simple academic task.^15,16^ We must remain aware of this generative process as we pass through thresholds and milestones from the perspectives of a student, supervisor and institution, all of which are used to monitor the doctoral progress. The doctoral journey in family medicine resembles the Hero’s Journey, divided into three acts: departure, initiation and return. The call to adventure arises as clinicians feel a stronger pull towards research, often motivated by a sense of purpose or recognition of systemic gaps in primary care. Crossing the threshold signifies the shift from practitioner to scholar, facing challenges such as organising study leave, balancing clinical responsibilities and managing the uncertainty of this new role. During trials and alliances, candidates face obstacles – such as funding setbacks, isolation and methodological difficulties – while drawing strength from supervisors, peers and digital tools. These experiences drive transformation, leading to shifts in identity from dependent learners to independent researchers. Ultimately, the return with wisdom represents not only the completion of a thesis but also a renewed commitment to improving health systems, mentoring others and advocating for evidence-based primary care.

Table 1 summarises the key insights from the authors’ journeys, aligned with the archetypes and early stages of the Hero’s Journey. Table 2 represents general stages or phases of a PhD, ranging from dreaming to doing to completing.

**TABLE 1:** Key insights for each step of the Hero’s Journey.

Archetypes and journey stages	Vignette 1	Vignette 2	Vignette 3	Vignette 4
Archetypal expressions	Orphan and Warrior, navigating isolation and institutional gaps with resilience	Wanderer and Altruist, balancing clinical service with academic purpose	Warrior and Caregiver, demonstrating perseverance and adaptability in the face of structural and personal challenges	Magician and Altruist, integrating resilience and strategic reflection to transform obstacles into opportunities for personal and scholarly development
Call to Adventure	I wanted to maintain my generalist clinical identity, so I pursued a PhD in epidemiology when no suitable speciality existed locally.	I was a newly qualified FP in a rural hospital, drawn to a national research project that aligned with my deeper purpose.	I always knew a PhD was part of my academic path, but I lacked mentorship and funding in family medicine.	I needed my PhD to contribute knowledge that could influence policy and address health inequities.
Crossing the Threshold	Transitioning from manager to student was tough; coursework and proposal development were overwhelming.	Negotiating a hybrid model to stay clinically active while doing research was my first major challenge.	I struggled to get study leave, manage family obligations, and secure research funding.	Managing fieldwork logistics and journal timelines added unexpected administrative weight.
Trials and Allies	My scholarship, committed supervisors and flexible programme helped me stay balanced and focused.	My supervisor, near-peer mentor and rural setting helped me stay grounded and reflective.	I leaned on tech tools, a study companion and online writing resources to overcome procrastination and isolation.	I built informal peer networks, set boundaries for self-care and stayed anchored by my ‘why’.

FP, family physician; PhD, Doctor of Philosophy.

**TABLE 2:** The doctoral journey mapped: dreaming, doing and completion.

Phase	Activities
Pre-PhD: The dreaming phase	This stage involves exploring motivations and expectations, selecting the right programme and supervisors and striking a balance between clinical work and academic objectives. Also, consider whether your topic is sufficiently original and broad, what qualities to seek in a supervisor and whether you need a co-supervisor. Are you ready for a 4–5-year commitment, and how will a PhD: Doctor of Philosophy benefit your career? A potential supervisor and academic institution will likewise assess the PhD from an educational perspective: does the candidate have prior scholarship, and will supporting them in obtaining a PhD help in developing an experienced researcher (progressing from emerging to established) who can supervise others and advance in discipline or field?
Year 1: Foundation of the doing phase	Learn how to learn. Doctoral students begin by learning the language of academia, developing a research concept note into a full proposal and building relationships and support networks. There are several opportunities for learning the skill sets required for the doctoral project.
Year 2: Deep dive and data	This stage involves data collection and analysis, navigating ethical and gatekeeper approvals and managing time, energy and imposter syndrome.
Year 3 and beyond – towards completion: Writing and identity	Students use writing for thinking, identity and preparing for defence and post-PhD life. Late-stage PhDs involve intense, deadline-driven writing and revision, with students often struggling when major revisions arise near submission, especially after a period of silence on feedback. These issues can negatively impact work quality and well-being. An early, structured feedback calendar can prevent this.

PhD, Doctor of Philosophy.

## Practical tips for success

We reflected on key tips (summarised in Box 5) that we would have offered our younger selves as we approached the threshold of commencing our PhD journey. Successfully navigating the PhD journey starts with *understanding your core motivation*. Even if your ‘why’ is not immediately clear, remaining receptive to discovering it is essential. Think of defining your rhumb line – a steady, guiding purpose that helps you stay grounded, especially when clinical work complements your research. A hybrid approach to balancing your ‘day job’ and PhD project work can help maintain continuity and focus. These include using the early morning slot, writing and working before usual hours, taking leave for dedicated writing and working or buying out time and splitting the week between part-time clinical and research work. *Establishing healthy boundaries and practising self-compassion* are essential for well-being.^17^

BOX 5 Key tips for your PhD journey.

Remember to review these key tips regularly during each phase of your PhD journey:

- Understand your core motivation.
- Establish healthy boundaries and practise self-compassion.
- Pursue scholarships, grants and institutional aid to address funding issues.
- Maintain clear communication and alignment with your supervisor and institution.
- Organise yourself: pacing and planning are essential, including setting milestone goals and utilising digital tools to monitor progress.
- Attend coursework (if offered), academic meetings and conferences, engage in critical reading and academic writing and keep a research journal.
- Build support networks and choose the right allies such as supervisors, peers and mentors.

Persistent challenges in many doctoral programmes include inconsistent funding disbursement and delays in supervisory feedback, which can lead to financial insecurity, slow progress and psychological stress. Students should *pursue scholarships, grants and institutional aid* to address funding issues. *Clear communication and alignment* through Memoranda of Understanding (MOUs) can ease supervisory pressures. Institutions should consider mechanisms to streamline scholarship payments, ensure timely supervisor feedback and build supervisory capacity at the doctoral level.

Remember, progress is rarely linear; pace, milestones and planning are essential. Globally, there is a trend towards a more structured and collaborative approach to doctoral education.^18^ Supervisors are increasingly working in teams, and doctoral students are more likely to receive targeted interventions at specific stages of their research. They also tend to have milestones marking their progress. Examples of increased structure include the addition of coursework, an examination at the end of the first or second year and a 7-year deadline to complete the study. Success, therefore, depends on careful *organisation*, such as *setting milestone goals and using digital tools to monitor progress*. Managing time effectively involves prioritising high-impact tasks using techniques such as compiling writing blocks and scheduled breaks, following the Pomodoro technique, a reliable and sustainable method developed in the 1980s.^19^

Academic growth is supported by *attending coursework* (*if offered*), *academic meetings and conferences, engaging in critical reading and maintaining a research journal*. Writing is often viewed as a source of negative emotions, such as frustration and confusion, which frequently dominate the doctoral writing experience. Collaborative writing enhances self-regulation, motivation, emotional well-being, writing quality and completion rates. The doctoral reading process is closely linked to effective writing. Academic and professional identity is formed through activities such as writing and attending academic meetings and conferences. The lack of curriculated support, such as coursework or departmental seminars, has been linked to South Africa’s high doctoral dropout rates.^18^ Common challenges for doctoral students include defining research problems and managing frustration with the scientific method during real-world research. Although doctoral students are often viewed as independent researchers in training, they may initially face challenges in unstructured situations because of issues with self-regulation. Unlike coursework, research tasks are frequently poorly defined, which can cause anxiety and confusion.^17^ When this occurs, reaching out to your supervisor for guidance can help you refocus and monitor your progress. Thriving in your PhD requires a holistic approach: maintaining methodological rigour, starting to write early, seeking constructive feedback, connecting with the scholarly community represented by your academic discipline and planning for your future career.

*Building support networks and selecting the right allies – such as supervisors, peers and mentors – is vital*. International trends favour more structured, collaborative doctoral methods, which may boost success.

## Conclusion: From dreamers to doers

Reflecting on our journeys, we offer lived insights to those contemplating a PhD, guiding them from dreaming to doing and to embarking on their own Hero’s Journey. In terms of reflexivity, we acknowledge the diversity of our backgrounds, disciplines and contexts. We are three male and one female scholar–practitioners, with varied timelines of PhD completion and professional trajectories across African academic and clinical settings. Our lived experiences shape our reflections on navigating our doctoral work while balancing service, teaching and personal commitments. We recognise that our positionality – as insiders within the discipline and as recent graduates – offers both proximity and hindsight. This dual lens enables us to speak candidly to the emotional, intellectual and structural dimensions of the PhD journey. By applying the Hero’s Journey Framework, we seek to honour the transformative essence of doctoral education while recognising the challenges and enabling factors that have shaped our paths. We strive to provide prospective candidates with a grounded, empathetic and contextually relevant guide, rooted in both collective wisdom and individual experience. While our insider perspectives offer depth, we acknowledge that our specific contexts shape our reflections and may not represent all doctoral experiences in Africa. In line with previous regional-level discussions, we propose forming a scholarly core of emerging and established researchers at departmental, national and regional levels to help strengthen the discipline of family medicine and raise its profile.^20^

A PhD journey is a challenging process, with each step a possible cause of anxiety – from uncertain beginnings to integrating work and discoveries. Despite the difficulties, it is a rewarding experience that makes a significant contribution to society and one’s disciplinary knowledge base. We hope this reflective essay inspires prospective candidates to begin their journey with confidence, clarity and a sense of community. Future candidates should see themselves not just as students, but as future leaders in family medicine and primary care.

The authors of this publication received research funding from the National Institute for Health and Care Research (NIHR) (Grant Number: NIHR158451) under the project ‘Improving the core functions of primary care in sub-Saharan Africa’. The terms of this arrangement have been reviewed and approved by the NIHR in accordance with its policy on objectivity in research. The author, I.K.B., serves as an editorial board member of this journal.

K.B.v.P. led the conceptualisation, design and drafting of the manuscript. K.B.v.P., M.M., A.T.L. and I.K.B. provided intellectual input and critical revisions.

This work was supported by the NIHR (Grant Number: NIHR158451) under the project ‘Improving the core functions of primary care in sub-Saharan Africa’.

The views and opinions expressed in this article are those of the authors and are the product of professional research. They do not necessarily reflect the official policy or position of any affiliated institution, funder, agency or that of the publisher. The authors are responsible for this article’s results, findings and content.
